# Whole genome sequencing snapshot of multi-drug resistant *Klebsiella pneumoniae* strains from hospitals and receiving wastewater treatment plants in Southern Romania

**DOI:** 10.1371/journal.pone.0228079

**Published:** 2020-01-30

**Authors:** Marius Surleac, Ilda Czobor Barbu, Simona Paraschiv, Laura Ioana Popa, Irina Gheorghe, Luminita Marutescu, Marcela Popa, Ionela Sarbu, Daniela Talapan, Mihai Nita, Alina Viorica Iancu, Manuela Arbune, Alina Manole, Serban Nicolescu, Oana Sandulescu, Adrian Streinu-Cercel, Dan Otelea, Mariana Carmen Chifiriuc

**Affiliations:** 1 National Institute for Infectious Diseases ‘Matei Bals’, Bucharest, Romania; 2 Institute of Biochemistry, Romanian Academy, Bucharest, Romania; 3 The Research Institute of the University of Bucharest, Bucharest, Romania; 4 Department of Botany and Microbiology, Faculty of Biology, University of Bucharest, Bucharest, Romania; 5 The National Institute of Research and Development for Biological Sciences, Bucharest, Romania; 6 University of Medicine and Pharmacy “Carol Davila”, Bucharest, Romania; 7 Department of Genetics, Faculty of Biology, University of Bucharest, Bucharest, Romania; 8 National Institute for R & D in Industrial Ecology (ECOIND), Bucharest, Romania; 9 Infectious Diseases Hospital Galati, Galati, Romania; 10 Faculty of Medicine and Pharmacy “Dunarea de Jos”, University of Galati, Galati, Romania; 11 Targoviste County Hospital, Targoviste, Romania; Ross University School of Veterinary Medicine, SAINT KITTS AND NEVIS

## Abstract

We report on the genomic characterization of 47 multi-drug resistant, carbapenem resistant and ESBL-producing *K*. *pneumoniae* isolates from the influent (I) and effluent (E) of three wastewater treatment plants (WWTPs) and from Romanian hospital units which are discharging the wastewater in the sampled WWTPs. The *K*. *pneumoniae* whole genome sequences were analyzed for antibiotic resistance genes (ARGs), virulence genes and sequence types (STs) in order to compare their distribution in C, I and E samples. Both clinical and environmental samples harbored prevalent and widely distributed ESBL genes, i.e. *bla*_SHV_, *bla*_OXA_, *bla*_TEM_ and *bla*_CTX M_. The most prevalent carbapenemase genes were *bla*_NDM-1_, *bla*_OXA-48_ and *bla*_KPC-2_. They were found in all types of isolates, while *bla*_OXA-162_, a rare *bla*_OXA-48_ variant, was found exclusively in water samples. A higher diversity of carbapenemases genes was seen in wastewater isolates. The aminoglycoside modifying enzymes (AME) genes found in all types of samples were *aac(6’)*, *ant(2'')Ia*, *aph(3')*, *aaD*, *aac(3)* and *aph(6)*. Quinolone resistance gene *qnrS1* and the multi-drug resistance *oqxA/B* pump gene were found in all samples, while *qnrD* and *qnrB* were associated to aquatic isolates. The antiseptics resistance gene *qacEdelta1* was found in all samples, while *qacE* was detected exclusively in the clinical ones. Trimethroprim-sulfamethoxazole (*dfrA*, *sul1* and *sul2*), tetracyclines (*tetA* and *tetD*) and fosfomycin (*fosA6*, known to be located on a transpozon) resistance genes were found in all samples, while for choramphenicol and macrolides some ARGs were detected in all samples (*catA1* and *catB3* / *mphA*), while other (*catA2*, *cmIA5* and *aac(6’)Ib* / *mphE* and *msrE*) only in wastewater samples. The rifampin resistance genes *arr2* and *3* (both carried by class I integrons) were detected only in water samples. The highly prevalent ARGs preferentially associating with aquatic *versus* clinical samples could ascribe potential markers for the aquatic (*bla*_SHV-145_, *qacEdelta1*, *sul1*, *aadA1*, *aadA2*) and clinical (*bla*_OXA-1_, *bla*_SHV-106_,*bla*_TEM-150_, *aac(3)Iia*, *dfrA14*, *oqxA10*; *oqxB17*,*catB3*, *tetD*) reservoirs of AR. Moreover, some ARGs (*oqxA10*; *bla*_SHV-145_; *bla*_SHV-100_, *aac(6')Il*, *aph(3')VI*, *armA*, *arr2*, *cmlA5*, *bla*_CMY-4_, *mphE*, *msrE*, *oqxB13*, *bla*_OXA-10_) showing decreased prevalence in influent *versus* effluent wastewater samples could be used as markers for the efficiency of the WWTPs in eliminating AR bacteria and ARGs. The highest number of virulence genes (75) was recorded for the I samples, while for E and C samples it was reduced to half. The most prevalent belong to three functional groups: adherence (*fim* genes), iron acquisition (*ent*, *fep*, *fyu*, *irp* and *ybt* genes) and the secretion system (*omp* genes). However, none of the genes associated with hypervirulent *K*. *pneumoniae* have been found. A total of 14 STs were identified. The most prevalent clones were ST101, ST219 in clinical samples and ST258, ST395 in aquatic isolates. These STs were also the most frequently associated with integrons. ST45 and ST485 were exclusively associated with I samples, ST11, ST35, ST364 with E and ST1564 with C samples. The less frequent ST17 and ST307 aquatic isolates harbored *bla*_OXA-162_, which was co-expressed in our strains with *bla*_CTX-M-15_ and *bla*_OXA-1_.

## Introduction

Antibiotic resistance (AR) is presently considered one of the most serious global public health threats, with the potential to become significantly more problematic by 2020 [1] due to globalization, environmental, social and demographic changes and health system capacity [2]. One of the priority topics endorsed by WHO (Word Health Organization) and JPIAMR (The Joint Programme Initiative on Antimicrobial Resistance) for tackling AR is to determine the role played by the environment in the selection and dissemination of AR [3, 4]. Wastewater treatment plants (WWTPs) have been recently suggested to be hotspots providing the perfect environment for the enrichment, recombination and selection of AR “super-bugs” which could eventually be discharged and subsequently impact adversely the environment and human health, thus highlighting the necessity for strategies of water quality improvement [5].

In 2018, a national consortium composed of the important Romanian institutions involved in water quality control and epidemiology of AR started the RADAR project, aiming to investigate the dynamics of AR and antibiotic resistance genes (ARGs) in ESKAPE pathogens (*Enterococcus faecium*, *Staphylococcus aureus*, *Klebsiella pneumoniae*, *Acinetobacter baumanii*, *Pseudomonas aeruginosa* and *Enterobacter* sp.) isolated from clinical and wastewater samples [6].

Here we report results of a RADAR sub-study aiming the genomic characterization of carbapenem resistant and extended spectrum β-lactamase (ESBL)-producing *K*. *pneumoniae* isolates from the influent and effluent of three WWTPs compared to clinical isolates obtained from Romanian hospital units which are discharging the wastewater in the sampled WWTP influent in order to evaluate the contribution of WWTPs to the AR reservoir and to reveal potential transmission between the water and clinical compartments. The main reasons to choose *K*. *pneumoniae* is the high prevalence of infections with *K*. *pneumoniae* strains resistant to carbapenems (22.5%), 3^rd^ generation cephalosporins (62.55%) and multidrug resistant—MDR (55.4%) reported in Romania [7]. Moreover, the hospital effluents are often released in the urban WWTP influent, increasing the risk of antibiotic resistant bacteria to be disseminated in the environment. However, the epidemiology of resistant *K*. *pneumoniae* clinical strains in WWTPs in Romania is currently unknown. Moreover, *K*. *pneumoniae* is a good indicator of the transmission between clinical and environmental AR reservoirs, being an ubiquitous microorganism found in soil, surface water and on plants [8], but also one of the most important Gram-negative opportunistic pathogens, frequently associated with both hospital and community acquired severe infections. [9, 10]; Moreover, *K*. *pneumoniae* could cumulate resistance (e.g. MDR, carbapenemase production) and hypervirulence (e.g. hypermucoviscosity) features could generate a new clinical crisis [11, 12, 13].

## Methodology

### Isolation and phenotypic characterization of *Klebsiella pneumoniae* strains

#### Sampling location

The wastewater samples were collected during December 2018 –June 2019 from three WWTPs and the clinical units discharging the hospital wastewater in the sampled WWTPs, located in Southern Romania: Bucharest (44.43225 N 26.10626 E), Galați (45.45 N 28.05 E) and Târgoviște  (44.92543 N 25.4567 E). The WWTP influent wastewaters have been sampled at locations of highly turbulent flow in order to ensure good mixing. The WWTP effluent samples were collected from downstream from all entering wastewater streams prior to discharge into the receiving waters. Permission was granted by the managers of the privately owned WWTPs.

The clinical strains have been collected from three clinical units, respectively the National Institute for Infectious Diseases ‘Matei Bals’, Bucharest, Romania (680 beds), Infectious Diseases Hospital Galați, Romania (160 beds) and Târgoviște County Hospital, Romania (Intensive Care, Infectious Diseases, Surgery Units) (1767 beds). The study was cleared by the local IRBs at all three clinical sites.

#### Isolation and characterization of *K. pneumoniae* strains

The analysed *K*. *pneumoniae* strains were isolated from influent and effluent water samples collected in sterile glass sample containers, transported to the laboratory at 5±3° C and processed within less than 24 hours. The water samples were diluted and filtered through 0.45 μm pore size membrane filters (Millipore, France), as described in SR EN ISO 9308-2/2014 (for coliform bacteria), using the following antibiotic-enriched media (BioMérieux, France): ChromID ESBL agar for extended spectrum beta-lactamases (ESBL)–producing *Enterobacteriaceae* and non-*Enterobacteriaceae* strains, ChromID OXA-48 agar and ChromID CARBA agar for carbapenemase (CRE)-producing *Enterobacteriaceae*. The resistant colonies obtained after cultivation at 37° C for 24 hours in aerobic conditions were subsequently inoculated on the selective media for the confirmation of the beta-lactam resistance phenotype. A total of 178 *K*. *pneumoniae* wastewater strains were recovered from the antibiotic-enriched media and identified using the MALDI-TOF-MS Bruker system: 96 from the influent and 82 from the effluent. The study also included 17 strains isolated during the same period from patients hospitalized in the clinical units discharging the wastewater in the sampled WWTPs. The antibiotic susceptibility profiles of *K*. *pneumoniae* strains were determined using the standard disc diffusion method according to The Clinical & Laboratory Standards Institute (CLSI) 2018 guidelines [14]. The antibiotics tested were: ampicillin (AMP), piperacillin (PRL), amoxicillin-clavulanic acid (AMC), aztreonam (ATM), meropenem (MEM), imipenem (IMP), ertapenem (ETP), cefuroxime (CXM), cefoxitin (FOX), ceftriaxone (CRO), cefepime (FEP), gentamicin (GEN), amikacin (AMK), tetracycline (TET), trimethoprim-sulfamethoxazole (SXT), ciprofloxacin (CIP).

#### Selection of *K*. *pneumoniae* strains for sequencing analyses

From the total of 195 *K*. *pneumoniae* water and clinical strains, 47 strains (of which 18 from influent, 16 from effluent and 13 from clinical settings) were selected for sequencing. The water strains were selected to include all three geographical areas, both influent and effluent strains and isolates recovered from all three chromogenic media, i.e. ChromID ESBL agar (8 strains), ChromID OXA-48 (10 strains) and ChromID CARBA (18 strains) (S1 Table). The 13 clinical strains were selected from individual ESKAPE isolates collected during one month prior to wastewater collection in the three hospitals. The clinical strains displaying highly similar or identical resistance phenotypes with the water strains isolated from the same geographical area were given priority.

### Next generation sequencing (NGS) setup

Bacterial DNA was extracted using DNeasy UltraClean Microbial Kit (Qiagen) and an in-house protocol based on mechanical and chemical bacterial lysis followed by DNA precipitation with ethanol. The Nextera DNA Flex Library Prep Kit (Illumina) was further used according to the manufacturer recommendations. Before sequencing, the DNA pool libraries were checked for optimal quality (2100 Bioanalyser, Agilent) and quantity (Qubit 4 Fluorimeter, Thermo Fisher Scientific). Forty-seven bacterial isolates were sequenced on the Miseq platform (Illumina) by using the paired-end shotgun strategy and Miseq reagent kit v.3 (600 cycles). This ensures the highest output of all MiSeq kits and generates sequences up to 300 bp long.

### Bioinformatics setup

*K*. *pneumoniae* whole genome sequences were analyzed using an NGS bioinformatics pipeline which runs on a Conda environment under Linux. Relevant information was extracted from NGS data by following the next steps: **1.** The raw MiSeq Illumina pair-end (PE) reads were trimmed of the adaptor sequences using BBDuk program from the BBTools suite [15]; **2.** The trimmed reads were assembled using SPAdes *de novo* assembler [16]; **3.** Average Nucleotide Identity (ANI) was applied using the FastANI program [17] on the resulting scaffolds from SPAdes, in order to double-check the species against the *K*. *pneumoniae* complete assembled reference genomes downloaded from NCBI; **4.** Antimicrobial resistance and virulence genes were identified in the *de novo* assembled scaffolds using the ABRicate program [18] to query the NCBI Bacterial Antimicrobial Resistance Reference Gene Database and the Virulence Factors database (VFDB), respectively. Plasmid replicon types were determined using the PlasmidFinder (implemented in ABRicate) [19]; **5.** Multilocus sequence typing (MLST) and the sample subtype (ST) were predicted using the MLST program [20]. Finally, reference mapping and BLAST searches of the contigs have been performed with Geneious Prime 2019.2.1 (https://www.geneious.com) [21].

The sets of virulent and resistant genes were further compared against the genes from the CARD [22] and VFDB [23] databases corresponding to *K*. *pneumoniae*.

The manipulation of the genomic information extracted from the MLST, resistance and virulence gene predictions, as well as plotting the resulting data was performed and computed in Excel, with the use of pivot tables and built-in functions.

The data have been filtered based on the extraction sites of the samples, on the number of wastewaters or clinical samples, on the MLST and based on the most important classes of antibiotic resistance and virulence genes.

The selected antimicrobial resistance genes (ARGs) to different antimicrobial agents were grouped in the following sets: beta-lactam (ESBLs and carbapenemases), aminoglycosides, other antimicrobial agents (trimethoprim-sulfamethoxazole, tetracyclines, chloramphenicol, fosfomycin, rifampicin and macrolides), quinolones and antiseptics.

Identification of integron presence was assessed by determination of integrase genes for class I, II and III integrons using BLAST tool (https://blast.ncbi.nlm.nih.gov/) [24], using *intl1* (Accession No: NC_019081), *intl2* (Accession No. NZ_CP025853.1:24029–24565) and *int3* (Accession No. NC_014356.1:c1521-481) as query sequences and the draft genome sequences as subjects.

The assembled sequences have been deposited in GenBank with Bioproject ID: PRJNA579879.

## Results

### Antibiotic susceptibility testing

Out of the total of 178 *K*. *pneumoniae* strains recovered from the antibiotic-enriched media: 96 from the influent and 82 from the effluent, 34 water strains (18 from influent and16 from effluent) were selected for whole genome sequencing. The water strains have been recovered from chromogenic media, i.e. ChromID ESBL agar (8 strains), ChromID OXA-48 (10 strains) and ChromID CARBA (18 strains) (S1 Table). The *in vitro* antibiotic susceptibility profiles of the sequenced *K*. *pneumoniae* isolates confirmed the expected resistance phenotypes of the strains recovered from the ChromID ESBL agar (i.e. resistance to at least 3^rd^ generation cephalosporins) and ChromID OXA-48 and ChromID CARBA (i.e. resistance to at least one carbapenem). A number of 13 clinical strains isolated from the three hospitals on culture media currently used in the respective clinical settings has been included in the study (S1 Table). The great majority of the strains selected for sequencing were MDR (S1 Table).

### Antimicrobial susceptibility profiles of the analysed strains

The antibiotic susceptibility assay of the tested strains has revealed that 92.30% of the clinical strains, and 87.5% and 82.3% respectively from the strains isolated from the WWTP effluent and influent were MDR. The clinical strains exhibited 92.30% resistance to AMP, PRL, ATM, FEP, and over 70% resistance to CXM, CIP, TET, SXT. The WWTP influent strains were 100% resistant to AMP, PRL and FEP and over 80% resistant to AMC, CXM, CRO, ATM, IMP, MEM, ETM, CIP, SXT, while the WWTP effluent strains were 100% resistant to AMP, SXT and over 80% resistant to PIP, AMC, CXM, FEP, ETP, ATM and CIP (S1 Table).

### Antimicrobial resistance genes (ARGs) distribution

The most prevalent **ARGs** detected in the 47 *K*. *pneumoniae* isolates selected for analysis (18from influent, 16 from effluent and 13 from clinical samples) were as follows:

- In **influent** samples, the ARGs with prevalence >50% (n = 12, in decreasing order) are those which confer resistance to aminoglycosides and beta-lactams: *ant(2'')Ia*, *qacEdelta1*, *sul1*, *aac(6')IId*, *aadA1*, *fosA*, *bla*_CTX-M-15_, *bla*_TEM-1_, *dfrA14*, *aph(3'')Ib*, *aph(6)Id*, *sul2*. Other ARG genes identified (n = 33) (with prevalence 25%-50%) were: *bla*_SHV187_, *oqxA10*, *oqxA*, *oqxB*, *catB3*, *bla*_OXA-1_, *fosA6*, *mphA*, *catA1*, *dfrA12*, *bla*_SHV-158_, *aadA2*, *bla*_SHV-145_, *aac(3)Iia*, *bla*_OXA-48_, *qnrS1*, *bla*_KPC-2_, *bla*_OXA-9_, *bla*_SHV-12_, *ble*, *bla*_NDM-1_, *catA2*, *bla*_SHV-100_, *aac(6')Il*, *aph(3')VI*, *armA*, *arr2*, *cmlA5*, *bla*_CMY-4_, *mphE*, *msrE*, *oqxB13*, *bla*_OXA-10_ (S2 Table).
- In **effluent** isolates, of the highly prevalent ARGs (n = 12), eight were similar to those found in the influent samples, i.e.: *ant(2'')Ia*, *qacEdelta1*, *sul1*, *aac(6')IId*, *aadA1*, *fosA*, *bla*_CTX-M-15_, *bla*_TEM-1_, while four were more frequently found in these isolates, comparatively to the influent ones, i.e.*oqxA*, *oqxB*, *fosA6*, *mphA*. On the other hand, the diversity of ARGs with prevalence between 25% and 50% (n = 15) is much more reduced compared to the influent set: *dfrA14*, *aph(3'')Ib*, *aph(6)Id*, *sul2*, *bla*_SHV-187_, *catB3*, *bla*_OXA-1_, *catA1*, *dfrA12*, *bla*_SHV-158_, *aadA2*, *aac(3)IIa*, *bla*_OXA-48_, *qnrS1*, *tet(A)* (S2 Table).
  The prevalence of some ARGs was significantly different in influent *versus* effluent wastewater samples, i.e.: it is much higher in influent as compared to effluent samples for *oqxA10* (44.4% in influent, 25% in effluent); *bla*_SHV-145_ (33.3% in influent, 6.3% in effluent); *bla*_SHV-100_, *aac(6')Il*, *aph(3')VI*, *armA*, *arr2*, *cmlA5*, *bla*_CMY-4_, *mphE*, *msrE*, *oqxB13*, *bla*_OXA-10_ (27.8% in influent, 6.3% in effluent). In the case of *tet(A)* prevalence is much more decreased in influent 5.6% as compared to 37.5% in effluent).
- In **clinical isolates**, the highly prevalent ARGs (> 50%) (n = 12) are slightly different, i.e.: *ant(2'')Ia*, *aac(6)IId*, *fosA*, *bla*_CTX-M-15_, *bla*_TEM-1_, *dfrA14*, *oqxA10*, *catB3*, *bla*_OXA-1_, *aac(3)Iia*, *oqxB17*, *bla*_SHV-106_, only four genes being similar to those found in the wastewater compartments. The diversity of ARGs with prevalence between 25% and 50% in the clinical samples (n = 10) is the lowest, compared with the water samples (S2 Table).

**Fig 1:**
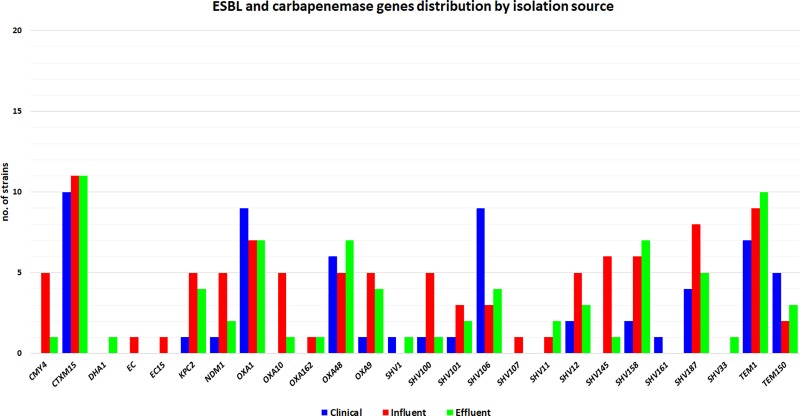
Beta-lactam resistance genes detected in the *K*. *pneumoniae* isolates from wastewater and the corresponding clinical settings.

The analysis of the β-lactamases encoding ARGs has shown that the most prevalent and widely distributed genes are *bla*_SHV_, *bla*_OXA_, *bla*_TEM_ and *bla*_CTXM_. In the clinical samples the most frequent were *bla*_CTX-M-15_, *bla*_OXA-1_, *bla*_SHV-106_, *bla*_TEM-1_, *bla*_OXA-48_ and *bla*_TEM-150_. In the influent samples, resistance to β-lactams is determined mainly by the presence of *bla*_CTX-M-15_, *bla*_OXA-1_, *bla*_OXA-48_, bla_OXA-10_, *bla*_NDM-1_, *bla*_CMY-4_, *bla*_SHV-145_, *bla*_TEM-1_. In the effluent samples, the most prevalent were *bla*_CTX-M-15_, *bla*_OXA-48_, *bla*_OXA-1_ and *bla*_TEM1_, *bla*_SHV-158_, *bla*_SHV-187_ and *bla*_KPC-2_. We noticed a high prevalence of *bla*_KPC-2_ in both effluent and influent isolates from one of the sampled geographical locations (Târgoviște). The analysis of carbapenemases encoding ARGs (CRGs) has shown a slightly higher diversity of CRGs in wastewater isolates, as compared to clinical ones. The most prevalent were *bla*_NDM-1_, *bla*_OXA-48_, *bla*_KPC-2_, which were found in all types of isolates, while *bla*_OXA-162_ was found exclusively in water samples from different geographical locations (Fig 1).

**Fig 2:**
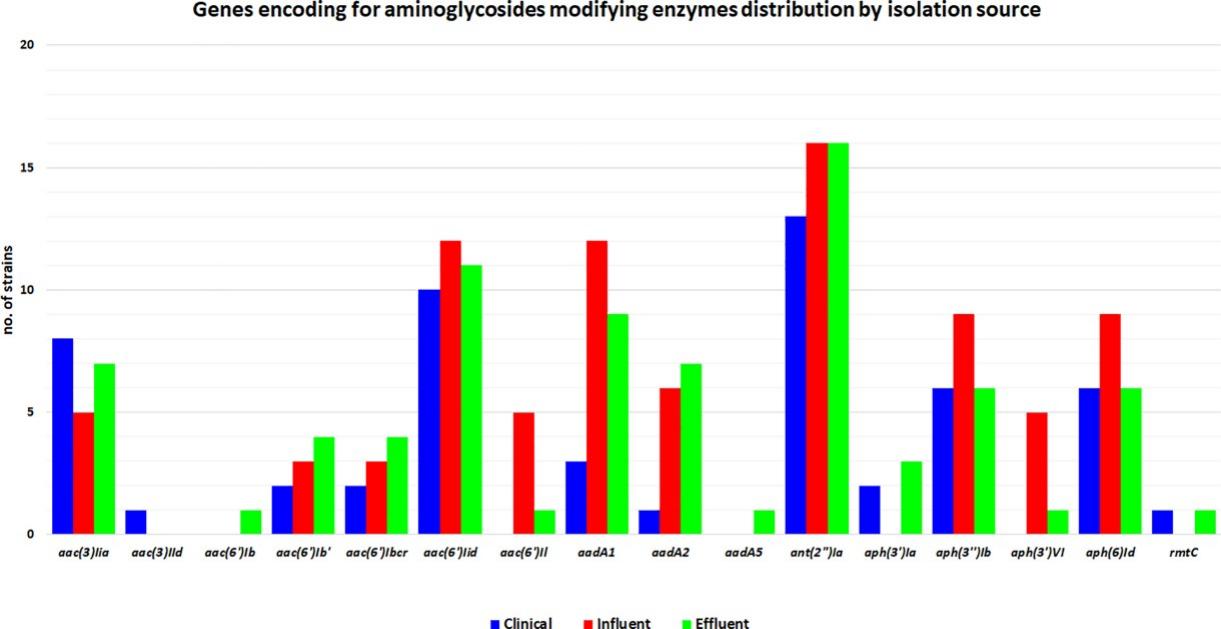
AME genes identified in the *K*. *pneumoniae* isolated from wastewater and clinical settings.

Fig 2 presents the most prevalent aminoglycoside modifying enzymes (AME) genes. The AME genes that were found in all types of samples were *aac(6’)*, *ant(2'')Ia*, *aph(3')*, *aaD*, *aac(3)* and *aph(6)*.

**Fig 3:**
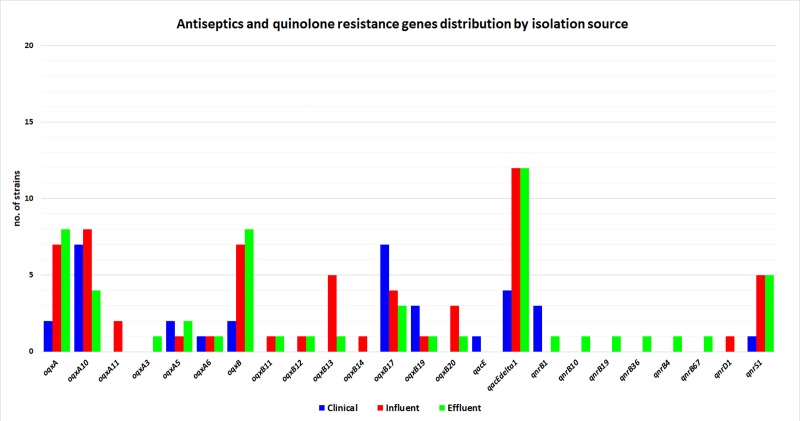
Identified quinolone and antiseptics resistance genes in wastewater and clinical samples.

Among the quinolone resistance genes, *qnrS1* was detected in all three types of samples from Bucharest, but was predominant in influent and effluent isolates. The *qnrD* (*qnrD1*) was detected exclusively in influent, while in the effluent samples the *qnrB* alleles were predominant. To these, the presence of the *oqxA/B* efflux pump, also responsible for resistance to antiseptics could explain the fluoroquinolone resistance. The most frequent antiseptics resistance genes were *oqxA*/*B*, followed by *qacEdelta1*, detected both in clinical and wastewater isolates and, with a much lower frequency, *qacE*, detected exclusively in clinical samples (Fig 3).

**Fig 4:**
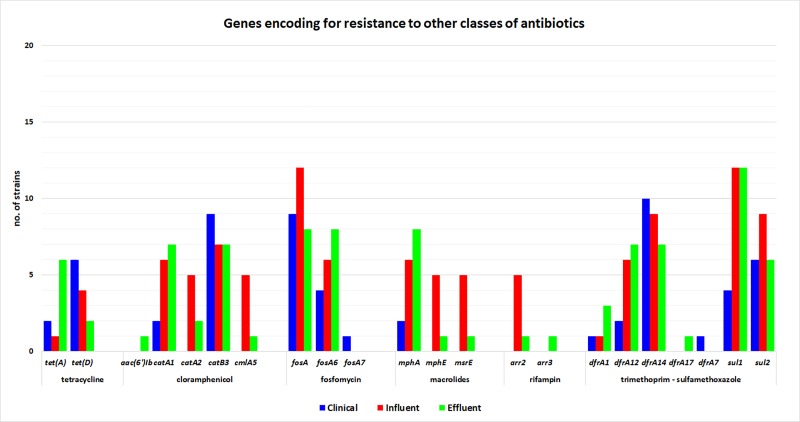
Resistance genes for other classes of antibiotics identified in aquatic and clinical samples.

ARGs for other classes of antimicrobial substances were also identified: for SXT were represented by *dfrA*, *sul1* and *sul2*, for tetracyclines by *tetA* and *D*, for choramphenicol by *catA1* and *catB3* (detected in all three types of samples), *cat2*, *cmIA5* and *aac(6’)Ib* (only in aquatic samples), for fosfomycin, by *fosA* (detected in all three types of samples), for macrolides by *mph(A)* (detected in all three types of samples) and *mphE* and *msrE* (only in wastewater samples) and for rifampin by *arr2* and *3*, detected only in water samples (Fig 4).

### Dynamics of ARGs among the clinical and environmental reservoirs

The following genes are of particular importance: *qacEdelta1*, *sul1* (both increasing from 30.8% in clinical strains to 66.7% / 75% in influent and effluent, respectively); *aadA1* (which increases from 23.1% in clinical strains to 66.7% / 56.3% in influent and effluent, respectively); *dfrA14* (which decreases from 76.9% in clinical strains to 50%/43.8% in influent and effluent, respectively); *oqxA10* (which decreases from 53.8% in clinical strains to 44.4% / 25% in influent and effluent, respectively); *catB3*, *bla*_OXA-1_ (both decrease from 69.2% in clinical strains to 38.9% / 43.8% in influent and effluent, respectively); *aadA2* (increases from 7.7% in clinical strains to 33.3% / 43.8% in influent and effluent, respectively); *bla*_SHV-145_ (which increases to 33.3% / 6.3% in influent and effluent, respectively, while missing in clinical strains); *aac(3)IIa* (decreases from 61.5% in clinical strains to 27.8% / 43.8% in influent and effluent, respectively); *oqxB17* (decreases from 53.8% in clinical strains to 22.2% / 18.8% in influent and effluent, respectively); *tet(D)* (which decreases from 46.2% in clinical strains to 22.2% / 12.5% in influent and effluent, respectively); *bla*_SHV-106_ (which decreases from 69.2% in clinical strains to 16.7% / 25% in influent and effluent, respectively) and *bla*_TEM-150_ (which decreases from 38.5% in clinical strains to 11.1% / 18.8% in influent and effluent, respectively).

### MLST distribution

Variations in MLST distribution among the three sample sets were observed and correlated both with geographical location and the sampling site (influent, effluent and clinical samples).

A total of 14 STs were identified in the *K*. *pneumoniae* strains analysed in this study. ST101 is the most prevalent clone (n = 13) closely followed by ST258 (n = 10). ST219 and ST395 have an equal prevalence (n = 5); ST307 (n = 3), ST1878 and ST17 (n = 2) have a low prevalence, while the following STs were identified only once: ST 219 like, ST45, ST485 only in influent (6%), ST11, ST35, ST364 only in effluent (6%) and ST1564 exclusively in the clinical isolates (S1 Fig).

**Fig 5:**
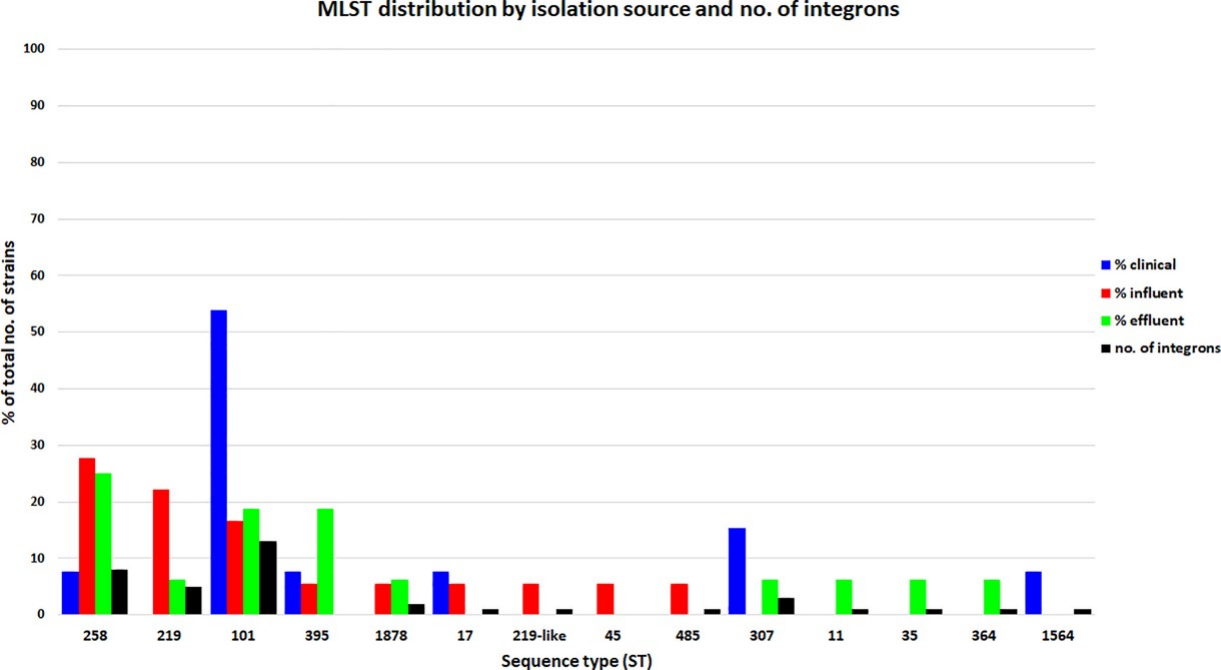
MLST and integrons distribution in the clinical, influent and effluent compartments.

The ST with the highest prevalence, ST101, was mostly identified in the clinical isolates (54%), while the second most prevalent one, ST258, was found in only 8% of the clinical isolates and 28% and respectively 25% of the influent and effluent isolates. Only ST395 was found in all sampling points, but identified most frequently in the effluent (19%). ST219 and ST1878 were isolated from influent (22%/6%) and effluent samples (6%/6%) while ST307 from clinical (15%) and effluent (6%) samples. The most frequently encountered STs, i.e. 101, 258 and 219 were also among the ones in which class I integron sequences were most frequently detected (Fig 5).

### Virulence genes distribution

A total number of 75 virulence genes were identified in the analyzed strains. Of these, the highly prevalent ones (n = 34, with prevalence over 50%) had a quite similar distribution among the samples with different isolation sources: *entA*, *entB*, *entE*, *entS*, *fepA*, *fepB*, *fepC*, *fepD*, *fepG*, *mgtB*, *mgtC*, *ompA*, *xcpA/pilD*, *xcpR*, *yagV/ecpE*, *yagW/ecpD*, *yagX/ecpC*, *yagY/ecpB*, *yagZ/ecpA*, *ykgK/ecpR*, *fimA*, *fimE*, *fyuA*, *irp1*, *irp2*, *ybtA*, *ybtE*, *ybtP*, *ybtQ*, *ybtS*, *ybtT*, *ybtU*, *ybtX*, *fimC* (S3 Table). The rest of the virulence genes (n = 41) had a prevalence of less than 25%.

These highly frequent cover 3 functional groups: adherence (*fim* genes); iron acquisition (*ent*, *fep*, *fyu*, *irp* and *ybt* genes); secretion system—T6SS-III(*omp* genes).

The *fimA*, *fyuA*, *irp1*, *irp2*, *ybtA*, *ybtE*, *ybtP*, *ybtQ*, *ybtS*, *ybtT*, *ybtU*, *ybtX* genes increased from 61.5% in clinical strains to 94.4–88.9% / 87.5% in influent and effluent, respectively, while *fimC* decreased from ~50% in clinical and influent to 25% in the effluent samples.

The highest number of virulence genes (n = 75) was found in the influent strains, while those detected in the effluent and clinical samples were lower by about 40% and 50% respectively (S3 Table).

## Discussions

Among the European countries, Romania is experiencing one of the highest rates of AR as revealed by the most recent data reported to EARSS (European Antimicrobial Resistance Surveillance System). The WWTPs are aquatic environments characterized by high-level selective pressure exerted by antibiotics and other chemical pollutants (such as heavy metals, drugs, metabolites); therefore, the comparative analysis of WWTPs and clinical isolates related in time and space could reveal trends of ARGs transmission and dissemination among the clinical and aquatic environment compartments. In this context, the RADAR national project has as a main goal to provide useful information and tools for bridging AR concerns in the hospital and the aquatic environment in Romania.

The main purpose of the current sub-study performed within the frame of RADAR project was to use WGS data for assessing the relatedness between clinical and environmental beta-lactam resistant *K*. *pneumoniae* isolates that could indicate the flow of ARGs from the hospital towards the environment and the reverse and thus providing a better understanding of the role of WWTP as an AR reservoir. The WGS data were obtained on *K*. *pneumoniae* isolates from three locations in southern Romania.

The strains isolated from Bucharest proved, as expected, a much higher rate of AR and diversity of ARGs and STs (S1 Fig) as compared to the other two geographical locations. This could be explained by the fact that the receiving WWTP from the capital city is much larger than those from Târgoviște and Galați. Moreover, Bucharest is by far more industrialized (therefore a higher selective pressure from pollutants can be expected inside WWTP, favouring the enrichment, recombination and selection of AR). The pathology in the Bucharest hospital unit is more diverse and more severe–cases from throughout the country are referred to the National Institute for Infectious Diseases “Matei Bals”.

The ESBL-producing *K*. *pneumoniae* strains represent a serious public health issue globally and locally [25].

The ESBL-positive *K*. *pneumoniae* strains harboured the most frequent and clinically relevant ESBL genes belonging to SHV, CTX-M, OXA and TEM families. The preponderance of *bla*_CTX-M-15_ and *bla*_SHV_ as the main ESBL genes in *K*. *pneumoniae* isolates confirms the results of other studies performed on Romanian clinical isolates, as well as the large worldwide distribution of these ESBL genes [26, 27, 28].

The most frequent carbapenemases genes (e.g. *bla*_OXA-48,_ *bla*_KPC-2,_ *bla*_NDM-1_ and *bla*_OXA-162_) found in the majority of the influent, effluent and clinical isolates are those reported as prevalent both in Romania and in other geographical areas [29]. Previous studies performed in Romania on carbapenem-non-susceptible *Klebsiella pneumoniae* clinical isolates have shown that *bla*_OXA-48_ was by far the most predominant genotype, followed by *bla*_NDM-1_ and *bla*_KPC-2_ [30, 31, 32]. The corresponding STs for the carbapenemase-producing isolates are: ST101 and ST258 (the most prevalent, as also reported in other studies including those performed on Romanian clinical strains by the authors of the present paper [11, 33], followed by ST11, ST17, ST219, ST307 and ST395). It’s worth mentioning that in two geographically different isolates from Bucharest (influent, ST17) and Galați (effluent, ST307) we have found *bla*_OXA-162_, a rare *bla*_OXA-48_ variant, differing by a single amino acid substitution (Thr213Ala), and which is usually co-expressed with ESBL genes (such as *bla*_TEM,_ *bla*_SHV,_ *bla*_CTX-M_), as in our case [34, 35]. This particular carbapenemase remains extremely rare, with few reports from Turkey [36], Germany [37], Hungary [38] and Greece [35]. In Greece [35], *bla*_OXA-162_ has been found to be co-expressed with *bla*_OXA-1_ and *bla*_DHA-1_ in ST11 isolates. It is interesting to note that in our study, of the two isolates containing *bla*_OXA-162_, one has also harboured *bla*_OXA-1_. Also, similar to the German and Hungarian studies, *bla*_OXA-162_ is co-expressed in both isolates with the *bla*_CTX-M-15_ [29, 38]. In contrast with other reports where *bla*_OXA-162_ was found in clinical isolates, in this study we have identified it in WWTP samples (both influent and effluent). To our knowledge, there is just one study that connects OXA-162 to the ST307 subtype [39] and there is none to connect it to ST17.

On the other hand, we verified the possibility that detected *bla*_OXA-162_gene in these two could be a sequencing artefact. Therefore, each assembled contig of these two isolates was further used as reference to map the corresponding raw Illumina PE reads onto. In the influent sample, the consensus sequence of *bla*_OXA-162_ gene was generated based on 8962 reads with a good coverage (mean = 269.6); In the effluent isolate, the contig was generated from 1483 reads having a satisfactory coverage (mean = 82.6).Performing comparative BLAST searches on both contigs resulted that both were identical (100% pairwise identity) with *K*. *pneumoniae* class D carbapenemases.

The most frequent STs in terms of frequency of isolation, geographical spreading and presence in both clinical and environmental compartments, i.e. 101, 258 and 219 were the most frequently associated with integrons. Class 1 integrons have been associated with the spread of resistance to antibiotics, disinfectants and heavy metals genes mainly in Gram-negative bacteria but also in Gram-positive strains. Furthermore, these mobile genetic elements represent a proxy for anthropogenic pollution [40].

Our study reveals for the first time the presence of carbapenemases-producing *K*. *pneumoniae* ST35, ST219, ST364, ST395, ST485 and ST1878 in wastewaters, of which ST395 has clinical importance, while ST35 and ST485 are sporadically related to clinical cases. The other STs, with major clinical significance, have already been described in wastewaters: ST11 [5, 41, 42, 43], ST17 [44], ST45 [45], ST101 [43, 46, 47, 48], ST258 [49], ST307 [45, 49, 50, 51], ST512 [52], and the minor clone ST1564 [45].

Fluoroquinolones resistance rates are increasing, especially in *Enterobacteriaceae*, due to their broad use for treating both Gram-negative and Gram-positive bacterial infections. Of the known mechanisms of resistance to quinolones overexpression of efflux pumps and plasmid mediated resistance *qnrB*, *D* and *S*, which protect DNA gyrase from quinolone inhibition were detected in our strains. It is well known that plasmid mediated quinolones resistance (PMQR) plasmids may also carry ESBL genes, including those harboured by our strains, i.e. *bla*_SHV_, *bla*_TEM_, *bla*_CTX-M_, *bla*_OXA_ and *bla*_KPC-2_, posing a great challenge for the treatment of the respective infections [53].

The most dominant aminoglycosides resistance gene was *aac(6')*, as also described in other studies [54], followed by *ant(2”)*, *aph(3”)* and *aadA*, either alone or in combination. The presence of these genes was higher in wastewater isolates, as compared to clinical ones, suggesting the important role of the aquatic environment as a reservoir of aminoglycosides resistance genes and the need for effective surveillance and strategies to reduce the selection pressure.

Similar to AME genes, trimethoprim-sulfamethoxazole resistance genes *dfrA*, *sul1* and *sul2* were also detected more frequently in wastewater samples, suggesting the need for careful surveillance of the aquatic reservoir for the presence of this type of resistance, particularly when taking into account that SXT is considered a low-cost alternative treatment by the Consortium on Resistance Against Carbapenems in *Klebsiella* and other *Enterobacteriaceae* (CRACKLE) [55, 56] and that in *K*. *pneumoniae*, *sul1* and *dfr* are highly prevalent in relation with class 1 integrons [57].

Similar to other studies, the most prevalent tetracyclines resistance genes were *tetA* and *tetD* [58].

Chloramphenicol resistance was mainly related to the presence of *catB* and *catA* genes, followed by *cmIA5*. Although *cmr* genes are reported by some studies as the most frequently found in clinical *K*. *pneumoniae* isolates [58], it was not present in the selected strains.

Although macrolides are not relevant for the treatment of Gram-negative infections, it has been suggested that commensal Gram-negative organisms may serve as a reservoir of ARGs that can be transferred to Gram-positive pathogens [59]. In our study, the most frequent macrolide ARG was *mphA*, followed by *mphE* and *msrE*.

Compared to other Gram-negative species, *K*. *pneumoniae* exhibits lower susceptibility to fosfomycin [60], and the most frequently reported mechanism is the production of the fosfomycin-inactivating enzyme f*osA* [61], which was identified in 99.7% of the *K*. *pneumoniae* genomes deposited in BLAST [62]. In our study *fosA6* was identified with high frequency both in clinical and wastewater isolates. Taking into account that *fosA6* is localized on a transposon and its transfer to a fosfomycin-resistant *E*. *coli* strain was already demonstrated [63], it can be suggested that *K*. *pneumoniae* isolates carrying the chromosomal *fosA*6 gene could serve as a reservoir of fosfomycin resistance in both clinical and aquatic environment.

The only rifampicin resistance mechanism harboured by our strains was the one linked to ADP-ribosyltransferase (*arr)* genes 2 and 3, revealed exclusively in the wastewater samples. The *arr-1*, *arr-2* and *arr-3* genes carried by class I integrons have been described in Gram-negative bacilli strains in Europe and Asia [64]. The *arr-2* gene which was the most frequently detected in the selected strains, particularly in influent samples, has been reported to be associated with several transposons and integrons in *K*. *pneumoniae* strains [65]. This raises the concern of mobilization and transmission of rifampicin resistance from *K*. *pneumoniae* strains to other clinically important pathogens.

The effect of biocidal agents used for disinfection to enhance cross-resistance to antibiotics has been highlighted in different reports. In the case of *K*. *pneumoniae*, occurrence of resistance to different antibiotics, including colistin, has been revealed after exposure to benzalkonium chloride [66] and chlorhexidine digluconate [67, 68]. Therefore, we have also followed the distribution of disinfectants resistance genes in the analyzed strains.

In our study, the *qacEdelta* and *qacE* genes have been more frequently associated with wastewater isolates, suggesting the selection of this type of resistance in the aquatic environment, probably due to the high selection pressure exerted by the presence of disinfectants. The *qacdeltaE* and *qacE* were isolated from a class I integron in the R751 plasmid, and were first documented in *K*. *pneumoniae* [69]. The *qacE* gene was, as expected, less encounteredsince it is predominantly associated with Gram-positive bacteria [70]. The most prevalent were the *oqxA/B* complex genes conferring resistance to multiple classes of antibiotics, but also to detergents and disinfectants. The *oqxA/B* complex can be located on chromosome and/or plasmids, flanked by *IS*26-like elements, posing thus a great risk for the public and environmental health, in terms of AMR horizontal transmission and selection of multiple-drug resistant phenotypes [71].

The highly prevalent ARGs preferentially associating with aquatic *versus* clinical samples could ascribe potential markers for the aquatic (i.e. *bla*_SHV-145_, *qacEdelta1*, *sul1*, *aadA1*, *aadA2*) and clinical (*bla*_OXA-1_, *bla*_SHV-106_, *bla*_TEM-150_, *aac(3)Iia*, *dfrA14*, *oqxA10*; *oqxB17*,*catB3*, *tetD*) reservoirs of AR.

Also, some ARGs (*oqxA10*; *bla*_SHV-145_; *bla*_SHV-100_, *aac(6')Il*, *aph(3')VI*, *armA*, *arr2*, *cmlA5*, *bla*_CMY-4_, *mphE*, *msrE*, *oqxB13*, *bla*_OXA-10_) showing a significantly decreased prevalence in influent *versus* effluent wastewater samples could be used as markers for the efficiency of the WWTPs in eliminating AR bacteria and ARGs. The higher *tetA* prevalence in the effluent as compared to influent could suggest that WWTPs favour the enrichment in tetracyclines resistant isolates.

Besides its resistance mechanisms, *K*. *pneumoniae* can also present different virulence factors, some of them responsible for the occurrence of hypervirulent *K*. *pneumoniae* severe infections [72]. The hypervirulence-associated factors are including capsular serotypes (K1 and K2), certain STs (ST 23 and CC 23), the virulence plasmid pLVPK and KPHP1208 pathogenicity island, as well as *RmpA* and *MagA* required for the mucoid phenotype and aerobactin. Despite the high number of virulence genes harboured by our strains, none of the genes associated with the hypervirulent *K*. *pneumoniae* genotype was detected.

The presented results contribute to enriching the knowledge of the epidemiological context of ESKAPE pathogens at national and European level, a major step in the implementation of reliable surveillance and actions plans. Whole genome sequencing is an essential tool that could provide fast and rich data on resistance genes, mobile genetic elements and virulence profiles, very useful for tracking AR reservoirs and transmission.

## Supporting information

S1 Table Antibiotic susceptibility testing results for the analyzed *K*. *pneumoniae* strains. (DOCX)

S2 Table ARG frequencies in clinical and aquatic samples. (DOCX)

S3 Table Virulence gene frequencies in clinical and aquatic samples. (DOCX)

S1 Fig MLST distribution among the three sampling sites. (TIF)

## References

[1] https://www.cdc.gov/drugresistance/biggest_threats.html.

[2] Suk JE, Semenza JC. Future infectious disease threats to Europe. Am J Public Health. 2011; 101(11):2068–79. 10.2105/AJPH.2011.300181 . 21940915 PMC3222407

[3] https://www.who.int/news-room/fact-sheets/detail/antibiotic-resistance.

[4] http://www.jpiamr.eu/activities/strategic-research-agenda/.

[5] Lepuschitz S, Schill S, Stoeger A, Pekard-Amenitsch S, Huhulescu S, Inreiter N et al Whole genome sequencing reveals resemblance between ESBL-producing and carbapenem resistant *Klebsiella pneumoniae* isolates from Austrian rivers and clinical isolates from hospitals. Sci Total Environ. 2019; 662:227–235. 10.1016/j.scitotenv.2019.01.179 . 30690357

[6] https://atlas.ecdc.europa.eu/public/index.aspx.

[7] https://ecdc.europa.eu/en/antimicrobial-resistance/surveillance-and-disease-data/data-ecdc.

[8] Khan FA, Hellmark B, Ehricht R, Söderquist B, Jass J. Related carbapenemase-producing *Klebsiella* isolates detected in both a hospital and associated aquatic environment in Sweden. Eur J Clin Microbiol Infect Dis. 2018; 37(12):2241–2251. 10.1007/s10096-018-3365-9 . 30171482

[9] Clegg S, Murphy CN. Epidemiology and Virulence of *Klebsiella pneumoniae*. Microbiol Spectr. 2016; 4(1):UTI–0005-2012 10.1128/microbiolspec.UTI-0005-2012 . 26999397

[10] Prokesch BC, TeKippe M, Kim J, Raj P, TeKippe EM, Greenberg DE. Primary osteomyelitis caused by hypervirulent *Klebsiella pneumoniae*. Lancet Infect Dis. 2016; 16(9):e190–e195. 10.1016/S1473-3099(16)30021-4 . 27402393

[11] Woodford N, Wareham DW, Guerra B, Teale C. Carbapenemase-producing *Enterobacteriaceae* and non-*Enterobacteriaceae* from animals and the environment: an emerging public health risk of our own making? J Antimicrob Chemother. 2014; 69(2):287–291. 10.1093/jac/dkt392 . 24092657

[12] Czobor I, Novais Ã, Rodrigues C, Chifiriuc MC, Mihăescu G, Lazăr V, et al Efficient transmission of IncFIIY and IncL plasmids and *Klebsiella pneumoniae* ST101 clone producing OXA-48, NDM-1 or OXA-181 in Bucharest hospitals. Int J Antimicrob Agents. 2016; 48: 223–4. 10.1016/j.ijantimicag.2016.05.004 27378198

[13] Lee CR, Lee JH, Park KS, Jeon JH, Kim YB, Cha CJ, et al Antimicrobial Resistance of Hypervirulent *Klebsiella pneumoniae*: Epidemiology, Hypervirulence-Associated Determinants, and Resistance Mechanisms. Front Cell Infect Microbiol.2017; 7:483 10.3389/fcimb.2017.00483 .13. 29209595 PMC5702448

[14] Clinical and Laboratory Standards Institute. 2018. Performance standards for antimicrobial susceptibility testing. 28th informational supplement..CLSI M100.

[15] https://jgi.doe.gov/data-and-tools/bbtools/.

[16] http://cab.spbu.ru/software/spades/.

[17] https://github.com/ParBLiSS/FastANI.

[18] https://github.com/tseemann/abricate.

[19] https://cge.cbs.dtu.dk/services/PlasmidFinder/.

[20] https://github.com/tseemann/mlst.

[21] https://www.geneious.com.

[22] https://card.mcmaster.ca.

[23] http://www.mgc.ac.cn/VFs/.

[24] Altschul SF, Gish W, Miller W, Myers EW, Lipman DJ. Basic local alignment search tool. J Mol Biol. 1990; 215(3):403–10. 10.1016/S0022-2836(05)80360-2 2231712

[25] Founou LL, Founou RC, Allam M, Ismail A, Djoko CF, Essack SY. Genome Sequencing of Extended-Spectrum β-Lactamase (ESBL)-Producing *Klebsiella pneumoniae* Isolated from Pigs and Abattoir Workers in Cameroon. Front. Microbiol. 2018; 9: 188, 10.3389/fmicb.2018.00188 . 29479347 PMC5811526

[26] Timofte D, Dan M, Maciuca IE, Ciucu L, Dabija ER, Guguianu E, et al Emergence of concurrent infections with colistin-resistant ESBL-positive *Klebsiella pneumoniae* and OXA-23-producing *Acinetobacter baumannii* sensitive to colistin only in a Romanian cardiac intensive care unit. Eur J Clin Microbiol Infect Dis. 2015; 34: 2069 10.1007/s10096-015-2453-3 26239064

[27] Licker M, Anghel A, Moldovan R, Hogea E, Muntean D, Horhat F, et al Genotype-phenotype correlation in multiresistant *Escherichia coli* and *Klebsiella pneumoniae* strains isolated in Western Romania. Eur Rev Med Pharmacol Sci. 2015; 19(10):1888–94. . 26044236

[28] Hristea A, Olaru ID, Adams-Sapper S, Riley LW. Characterization of ESBL-producing *Escherichia coli* and *Klebsiella pneumoniae* from bloodstream infections in three hospitals in Bucharest, Romania: a preliminary study. Infect Dis (Lond). 2015; 47(1):46–51. 10.3109/00365548.2014.959043 . 25365029

[29] Becker L, Kaase M, Pfeifer Y, Fuchs S, Reuss A, von Laer A, et al Genome-based analysis of Carbapenemase-producing *Klebsiella pneumoniae* isolates from German hospitalpatients, 2008–2014. Antimicrob Resist Infect Control. 2018; 7:62 10.1186/s13756-018-0352-y . 29744043 PMC5930415

[30] Lixandru BE, Cotar AI, Străuț M, Usein CR, Cristea D, Ciontea S, et al Carbapenemase-Producing *Klebsiella pneumoniae* in Romania: A Six-Month Survey. PLoS One. 2015; 10(11):e0143214 10.1371/journal.pone.0143214 . 26599338 PMC4658179

[31] Pirii LE, Friedrich AW, Rossen JWA, Vogels W, Beerthuizen GIJM, Nieuwenhuis MK, et al Extensive colonization with carbapenemase-producing microorganisms in Romanian burn patients: infectious consequences from the Colectiv fire disaster. Eur J Clin Microbiol Infect Dis. 2018; 37(1):175–183. 10.1007/s10096-017-3118-1 . 29063446 PMC5748401

[32] Székely E, Damjanova I, Jánvári L, Vas KE, Molnár S, Bilca DV, et al First description of *bla*_NDM-1_, *bla*_OXA-48_, *bla*_OXA-181_ producing *Enterobacteriaceae* strains in Romania. Int J Med Microbiol. 2013; 303(8):697–700. 10.1016/j.ijmm.2013.10.001 . 24183483

[33] David S, Reuter S, Harris SR, Glasner C, Feltwell T, Argimon S et al Epidemic of carbapenem-resistant Klebsiella pneumoniae in Europe is driven by nosocomial spread. Nat Microbiol. 2019; 2058–5276. 10.1038/s41564-019-0492-8 . 31358985 PMC7244338

[34] Ghafourian S, Sadeghifard N, Soheili S, Sekawi Z. Extended Spectrum Beta-lactamases: Definition, Classification and Epidemiology. Curr Issues Mol Biol. 2015; 17:11–21. . 24821872

[35] Voulgari E, Poulou A, Dimitroulia E, Politi L, Ranellou K, Gennimata V et al Emergence of OXA-162 Carbapenemase- and DHA-1 AmpCCephalosporinase-Producing Sequence Type 11 *Klebsiella pneumoniae* Causing Community-Onset Infection in Greece. Antimicrob Agents Chemother. 2015; 60(3):1862–4. 10.1128/AAC.01514-15 . 26666930 PMC4775966

[36] Kasap M, Torol S, Kolayli F, Dundar D, Vahaboglu H. OXA-162, a novel variant of OXA-48 displays extended hydrolytic activity towards imipenem, meropenem and doripenem. J Enzyme Inhib Med Chem. 2013; 28(5):990–6. 10.3109/14756366.2012.702343 . 22845331

[37] Pfeifer Y, Schlatterer K, Engelmann E, Schiller RA, Frangenberg HR, Stiewe D, et al Emergence of OXA-48-type carbapenemase-producing *Enterobacteriaceae* in German hospitals. Antimicrob Agents Chemother. 2012; 56(4):2125–8. 10.1128/AAC.05315-11 . 22290940 PMC3318349

[38] Jánvári L, Damjanova I, Lázár A, Rácz K, Kocsis B, Urbán E, et al Emergence of OXA-162-producing *Klebsiella pneumoniae* in Hungary. Scand J Infect Dis. 2014; 46(4):320–4. 10.3109/00365548.2013.879993 . 24552581

[39] Al-Baloushi AE, Pál T, Ghazawi A, Sonnevend A. Genetic support of carbapenemases in double carbapenemase producer *Klebsiella pneumoniae* isolated in the Arabian Peninsula. ActaMicrobiolImmunol Hung. 2018; 65(2):135–150. 10.1556/030.65.2018.005 . 29471690

[40] Gillings MR, Gaze WH, Pruden A, Smalla K, Tiedje JM, Zhu YG. Using the class 1 integron-integrase gene as a proxy for anthropogenic pollution. ISME J. 2015;9(6):1269–1279. 10.1038/ismej.2014.226 . 25500508 PMC4438328

[41] Sekizuka T, Yatsu K, Inamine Y, Segawa T, Nishio M, Kishi N, et al Complete Genome Sequence of a *bla*KPC-2-Positive *Klebsiella pneumoniae* Strain Isolated from the Effluent of an Urban Sewage Treatment Plant in Japan. mSphere. 2018; 19:3(5). pii: e00314–18. 10.1128/mSphere.00314-18 . 30232165 PMC6147131

[42] Jin L, Wang R, Wang X, Wang Q, Zhang Y, Yin Y, et al Emergence of mcr-1 and carbapenemase genes in hospital sewage water in Beijing, China. Antimicrob Chemother. 2018; 73(1):84–87. 10.1093/jac/dkx355 . 29040585

[43] Obasi A, Nwachukwu S, Ugoji E, Kohler C, Göhler A, Balau V, et al Extended-Spectrum β-Lactamase-Producing *Klebsiella pneumoniae* from Pharmaceutical Wastewaters in South-Western Nigeria. Microb Drug Resist. 2017; 23(8):1013–1018. 10.1089/mdr.2016.0269 . 28375698

[44] Alouache S, Estepa V, Messai Y, Ruiz E, Torres C, Bakour R. Characterization of ESBLs and associated quinolone resistance in *Escherichia coli* and *Klebsiella pneumoniae* isolates from an urban wastewater treatment plant in Algeria. Microb Drug Resist. 2014; 20(1):30–8. 10.1089/mdr.2012.0264 . 23952363

[45] Sghaier S, Abbassi MS, Pascual A, Serrano L, Díaz-De-Alba P, Said MB, et al Extended-spectrum β-lactamase-producing *Enterobacteriaceae* from animal origin and wastewater in Tunisia: first detection of O25b-B2(3)-CTX-M-27-ST131 Escherichia coli and CTX-M-15/OXA-204-producing Citrobacter freundii from wastewater. J Glob Antimicrob Resist. 2019; 17:189–194. 10.1016/j.jgar.2019.01.002 . 30639890

[46] Mahon BM, Brehony C, Cahill N, McGrath E, O'Connor L, Varley A, et al Detection of OXA-48-like-producing *Enterobacterales* in Irish recreational water. Sci Total Environ. 2019; 690:1–6. 10.1016/j.scitotenv.2019.06.480 . 31299565

[47] Anssour L, Messai Y, Estepa V, Torres C, Bakour R. Characteristics of ciprofloxacin-resistant *Enterobacteriaceae* isolates recovered from wastewater of an Algerian hospital. J Infect Dev Ctries. 2016; 10(7):728–34. 10.3855/jidc.6727 . 27482804

[48] Ben Said L, Jouini A, Alonso CA, Klibi N, Dziri R, Boudabous A, et al Characteristics of extended-spectrum β-lactamase (ESBL)- and pAmpC beta-lactamase-producing *Enterobacteriaceae* of water samples in Tunisia. SciTotal Environ. 2016; 550:1103–1109. 10.1016/j.scitotenv.2016.01.042 . 26871556

[49] Caltagirone M, Nucleo E, Spalla M, Zara F, Novazzi F, Marchetti VM, et al Occurrence of Extended Spectrum β-Lactamases, KPC-Type, and MCR-1.2-Producing *Enterobacteriaceae* from Wells, River Water, and Wastewater Treatment Plants in Oltrepò Pavese Area, Northern Italy. Front Microbiol. 2017; 8:2232 10.3389/fmicb.2017.02232 . 29176971 PMC5687051

[50] Ekwanzala MD, Dewar JB, Kamika I, Momba MNB. Tracking the environmental dissemination of carbapenem-resistant *Klebsiella pneumoniae* using whole genome sequencing. Sci Total Environ. 2019; 691:80–92. 10.1016/j.scitotenv.2019.06.533 . 31319261

[51] Dropa M, Lincopan N, Balsalobre LC, Oliveira DE, Moura RA, Fernandes MR, et al Genetic background of novel sequence types of CTX-M-8- and CTX-M-15-producing *Escherichia coli* and *Klebsiella pneumoniae* from public wastewater treatment plants in São Paulo, Brazil. Environ Sci Pollut Res Int. 2016; 23(5):4953–8. 10.1007/s11356-016-6079-5 . 26782324

[52] Perilli M, Bottoni C, Pontieri E, Segatore B, Celenza G, Setacci D et al Emergence of *bla*_KPC-3_-Tn4401a in *Klebsiella pneumoniae* ST512 in the municipal wastewater treatment plant and in the university hospital of a town in central Italy. J Glob Antimicrob Resist. 2013;1(4):217–220. 10.1016/j.jgar.2013.07.002 . 27873616

[53] Paterson DL, Mulazimoglu L, Casellas JM, Ko WC, Goossens H, Von Gottberg A, et al Epidemiology of ciprofloxacin resistance and its relationship to extended-spectrum beta-lactamase production in *Klebsiella pneumoniae* isolates causing bacteremia. Clin Infect Dis. 2000;30(3):473–8. 10.1086/313719 . 10722430

[54] Nasiri G, Peymani A, Farivar TN, Hosseini P. Molecular epidemiology of aminoglycoside resistance in clinical isolates of Klebsiella pneumoniae collected from Qazvin and Tehran provinces, Iran. Infect Genet Evol. 2018; 64:219–224. 10.1016/j.meegid.2018.06.030 . 29964191

[55] van Duin D, Perez F, Rudin SD, Cober E, Hanrahan J, Ziegler J, et al Surveillance of carbapenem-resistant *Klebsiella pneumoniae*: tracking molecular epidemiology and outcomes through a regional network. Antimicrob Agents Chemother. 2014; 58(7): 4035–41. 10.1128/AAC.02636-14 . 24798270 PMC4068524

[56] Luterbach CL, Boshe A, Henderson HI, Cober E, Richter SS, Salata RA, et al The Role of Trimethoprim/Sulfamethoxazole in the treatment of infections caused by carbapenem-resistant *Enterobacteriaceae*. Open Forum Infect Dis. 2018; 6(1):ofy351 10.1093/ofid/ofy351 30631796 PMC6324543

[57] Shin HW, Lim J, Kim S, Kim J, Kwon GC, Koo SH. Characterization of trimethoprim-sulfamethoxazole resistance genes and their relatedness to class 1 integron and insertion sequence common region in Gram-negative bacilli. J Microbiol Biotechnol. 2015; 25(1):137–42. 10.4014/jmb.1409.09041 . 25348695

[58] Taitt CR, Leski TA, Erwin DP, Odundo EA, Kipkemoi NC, Ndonye JN, et al Antimicrobial resistance of *Klebsiella pneumoniae* stool isolates circulating in Kenya. PLoS ONE.2017; 12(6): e0178880 10.1371/journal.pone.0178880 . 28575064 PMC5456380

[59] Nguyen MCP, Woerther P-L, Bouvet M, Andremont A, Leclercq R, Canu A. *Escherichia coli* as reservoir for macrolide resistance genes. Emerg Infect Dis. 2009; 15:1648–1650. 10.3201/eid1510.090696 19861064 PMC2866414

[60] Vardakas KZ, Legakis NJ, Triarides N, Falagas ME. Susceptibility of contemporary isolates to fosfomycin: a systematic review of the literature. Int J Antimicrob Agents. 2016; 47(4):269–85. 10.1016/j.ijantimicag.2016.02.001 . 27013000

[61] Falagas ME, Vouloumanou EK, Samonis G, Vardakas KZ. Fosfomycin. Clin Microbiol Rev. 2016; 29(2): 321–47. 10.1128/CMR.00068-15 . 26960938 PMC4786888

[62] Ito R, Mustapha MM, Tomich AD, Callaghan JD, McElheny CL, Mettus RT, et al Widespread Fosfomycin Resistance in Gram-Negative Bacteria Attributable to the Chromosomal fosA Gene. MBio. 2017; 8(4):e00749–17. 10.1128/mBio.00749-17 . 28851843 PMC5574708

[63] Guo Q, Tomich AD, McElheny CL, Cooper VS, Stoesser N, Wang M, et al Glutathione-S-transferase FosA6 of *Klebsiella pneumoniae* origin conferring fosfomycin resistance in ESBL-producing Escherichia coli. J Antimicrob Chemother. 2016; 71(9):2460–5. 10.1093/jac/dkw177 . 27261267 PMC4992852

[64] Gadou V, Guessennd NK, Toty AA, Konan F, Ouattara MB, Dosso M, et al Molecular Detection of the Arr-2 Gene in *Escherichia coli* and *Klebsiella pneumoniae* Resistant to Rifampicin in Abidjan, Côte D'Ivoire. Microbiol Res J Int. 2018; 23(4): 1–8, Article no.MRJI.40552.

[65] Arlet G, Nadjar D, Herrmann JL, Donay JL, Rouveau M, Lagrange PH, et al Plasmid-mediated rifampin resistance encoded by an arr-2-like gene cassette in *Klebsiella pneumoniae* producing an AAC1 class C β-lactamase. Antimicrob Agents Chemother. 2001; 45:2971–2972. 10.1128/AAC.45.10.2971-2972.2001 . 11583008 PMC90768

[66] Gadea R., Fernandez Fuentes M.A., Perez Pulido R., Galvez A., Ortega E. Effects of exposure to quaternary-ammonium-based biocides on antimicrobial susceptibility and tolerance to physical stresses in bacteria from organic foods. Food Microbiol. 2017; 63: 58–71. 10.1016/j.fm.2016.10.037 . 28040182

[67] Kampf G. Biocidal Agents Used for Disinfection Can Enhance Antibiotic Resistance in Gram-Negative Species. Antibiotics (Basel). 2018; 7(4): pii: E110 10.3390/antibiotics7040110 . 30558235 PMC6316403

[68] Zhang Y, Zhao Y, Xu C, Zhang X, Li J, Dong G, et al Chlorhexidine exposure of clinical *Klebsiella pneumoniae* strains leads to acquired resistance to this disinfectant and to colistin. Int J Antimicrob Agents. 2019; 53(6): 864–867. 10.1016/j.ijantimicag.2019.02.012 . 30818000

[69] Vijayakumara R, Sandle T, Al-Aboody MS, Al Fonaisan MK, Alturaiki W, Mickymaray S, et al Distribution of biocide resistant genes and biocides susceptibility in multidrug-resistant *Klebsiella pneumoniae*, Pseudomonas aeruginosa and *Acinetobacter baumannii*—A first report from the Kingdom of Saudi Arabia. J Infect Public Heal. 2018; 11(6): 812–816. . 10.1016/j.jiph.2018.05.011 29907439

[70] Wassenaar TM, Ussery D, Nielsen LN, Ingmer H. Review and phylogenetic analysis of qac genes that reduce susceptibility to quaternary ammonium compounds in *Staphylococcus* species. Eur J Microbiol Immunol. 2015; 5 (1): 44–61. 10.1556/EUJMI-D-14-00038 . 25883793 PMC4397847

[71] Li J, Zhang H, Ning J, Sajid A, Cheng G, Yuan Z, Hao H. The nature and epidemiology of OqxAB, a multidrug efflux pump. Antimicrob Resist Infect Control. 2019; 8:44 10.1186/s13756-019-0489-3 . 30834112 PMC6387526

[72] Lee CR, Lee JH, Park KS, Jeon JH, Kim YB, Cha CJ, et al Antimicrobial Resistance of Hypervirulent *Klebsiella pneumoniae*: Epidemiology, Hypervirulence-Associated Determinants, and Resistance Mechanisms. Front Cell Infect Microbiol. 2017; 7:483 10.3389/fcimb.2017.00483 . 29209595 PMC5702448

