# Supplementary material for: Whole genome sequencing snapshot of multi-drug resistant Klebsiella pneumoniae strains from hospitals and receiving wastewater treatment plants in Southern Romania
Source: PLoS One. 2020 Jan 30;15(1):e0228079. doi: 10.1371/journal.pone.0228079 (PMC6992004; doi:10.1371/journal.pone.0228079)
Supplement: S1 Table — (DOCX) [file pone.0228079.s001.docx]

**Supplementary Table 1.** Antibiotic susceptibility testing results for the analysed *K. pneumoniae* strains

| **Sampling point** | **Location** | **ID NGS** | **ST** | Selective media | **Antibiotic Susceptibility** | | | | | | | | | | | | | | | | | | |
| --- | --- | --- | --- | --- | --- | --- | --- | --- | --- | --- | --- | --- | --- | --- | --- | --- | --- | --- | --- | --- | --- | --- | --- |
|  |  |  |  |  | **AMP** | **PRL** | **AMC** | **CXM** | **FOX** | **CRO** | **CTX** | **KZ** | **FEP** | **ETP** | **IMP** | **MEM** | **ATM** | **AK** | **CN** | **TET** | **CIP** | **SXT** | **MDR** |
| **Influent** | Bucharest | 21 | 219-like | ChromID OXA-48 | R | R | R | R | R | R | - | - | R | R | R | R | R | R | R | R | R | R | + |
|  | Bucharest | 22 | 101 | ChromID OXA-48 | R | R | R | R | R | R | - | - | R | R | R | R | R | R | R | R | R | R | + |
|  | Bucharest | 24 | 485 | ChromID ESBL | R | R | R | R | S | R | - | - | R | R | S | R | R | S | R | S | R | R | + |
|  | Bucharest | 29 | 101 | ChromID CARBA | R | R | R | R | R | R | - | - | R | R | R | R | S | R | R | R | R | R | + |
|  | Bucharest | 30 | 219 | ChromID CARBA | R | R | R | R | R | R | - | - | R | R | R | R | R | R | R | S | R | R | + |
|  | Bucharest | 31 | 219 | ChromID OXA-48 | R | R | R | R | R | R | - | - | R | R | R | R | R | R | R | S | R | R | + |
|  | Bucharest | 32 | 17 | ChromID OXA-48 | R | R | R | - | S | R | - | - | R | R | R | R | R | S | R | S | S | S | - |
|  | Bucharest | 34 | 101 | ChromID CARBA | R | R | R | R | R | R | - | - | R | R | R | R | R | R | R | R | R | R | + |
|  | Bucharest | 35 | 219 | ChromID CARBA | R | R | R | R | R | R | - | - | R | R | R | R | R | R | R | R | R | R | + |
|  | Galati | 44 | 45 | ChromID ESBL | R | R | S | R | S | R | - | R | R | S | S | S | R | S | - | S | S | S | - |
|  | Bucharest | 68 | 1878 | ChromID ESBL | R | R | S | R | S | - | R | R | R | S | S | S | R | S | - | S | S | S | - |
|  | Bucharest | 69 | 395 | ChromID OXA-48 | R | R | R | R | R | - | R | R | R | R | R | R | R | R | - | R | R | R | + |
|  | Bucharest | 74 | 258 | ChromID CARBA | R | R | R | R | R | - | R | R | R | R | R | R | R | R | - | S | R | R | + |
|  | Targoviste | 99 | 258 | ChromID CARBA | R | R | R | R | R | R | - | R | R | R | R | R | R | R | S | S | R | R | + |
|  | Targoviste | 100 | 258 | ChromID CARBA | R | R | R | R | R | R | - | R | R | R | R | R | R | R | S | S | R | R | + |
|  | Targoviste | 101 | 258 | ChromID CARBA | R | R | R | R | R | R | - | R | R | R | R | R | R | R | S | S | R | R | + |
|  | Targoviste | 102 | 258 | ChromID CARBA | R | R | R | R | R | R | - | R | R | R | R | R | R | R | S | S | R | R | + |
|  | % of resistant strains /antibiotic | | | | 100 | 100 | 88.23 | 94.11 | 76.47 | 82.35 | 17.64 | 47.05 | 100 | 88.23 | 82.35 | 88.23 | 94.11 | 76.47 | 52.94 | 35.29 | 82.35 | 82.35 | 82.35 |
| **Effluent** | Bucharest | 19 | 219 | ChromID CARBA | R | R | R | R | R | R | - | - | R | R | R | R | R | R | R | S | R | R | + |
|  | Bucharest | 20 | 11 | ChromID ESBL | R | R | R | - | S | R | - | - | R | R | S | S | R | S | S | S | S | R | - |
|  | Bucharest | 23 | 101 | ChromID CARBA | R | R | R | R | R | R | - | - | R | R | R | R | R | R | R | R | R | R | + |
|  | Bucharest | 26 | 101 | ChromID CARBA | R | R | R | R | S | S | - | - | S | R | R | R | S | S | R | R | R | R | + |
|  | Bucharest | 28 | 364 | ChromID ESBL | R | - | R | R | S | R | - | - | R | R | S | S | R | R | S | S | R | R | + |
|  | Galati | 50 | 307 | ChromID CARBA | R | R | R | R | R | R | - | R | R | R | R | R | R | S | - | S | R | R | + |
|  | Bucharest | 76 | 258 | ChromID ESBL | R | R | R | R | R | - | R | R | R | R | R | R | R | R | - | S | R | R | + |
|  | Bucharest | 77 | 1878 | ChromID ESBL | R | R | S | R | S | - | R | R | R | S | S | S | R | S | - | S | S | R | - |
|  | Bucharest | 78 | 395 | ChromID OXA-48 | R | R | R | R | R | - | R | R | R | R | R | R | R | R | - | R | R | R | + |
|  | Bucharest | 79 | 395 | ChromID OXA-48 | R | R | R | R | R | - | R | R | R | R | R | R | R | R | - | R | R | R | + |
|  | Bucharest | 80 | 395 | ChromID OXA-48 | R | R | R | R | R | - | R | R | R | R | R | R | S | R | - | R | R | R | + |
|  | Bucharest | 82 | 101 | ChromID CARBA | R | R | R | R | R | - | R | R | R | R | R | R | S | R | - | R | R | R | + |
|  | Targoviste | 109 | 35 | ChromID ESBL | R | R | R | R | S | R | - | R | R | S | S | S | R | S | R | R | R | R | + |
|  | Targoviste | 113 | 258 | ChromID CARBA | R | R | R | R | R | R | - | R | R | R | R | R | R | R | S | S | R | R | + |
|  | Targoviste | 114 | 258 | ChromID CARBA | R | R | R | R | R | R | - | R | R | R | R | R | R | R | S | S | R | R | + |
|  | Targoviste | 115 | 258 | ChromID CARBA | R | R | R | R | R | R | - | R | R | R | R | R | R | R | S | S | R | R | + |
|  | % of resistant strains/antibiotic | | | | 100 | 93.75 | 93.75 | 93.75 | 68.75 | 56.25 | 37.5 | 68.75 | 93.75 | 87.5 | 75 | 75 | 81.25 | 68.75 | 25 | 43.75 | 87.5 | 100 | 87.5 |
| **Clinical** | Bucharest | 36 | 101 | - | R | R | R | R | R | R | - | - | R | R | S | S | R | R | R | R | R | R | + |
|  | Bucharest | 37 | 101 | - | R | R | S | R | S | R | - | - | R | S | S | S | R | R | R | R | R | R | + |
|  | Bucharest | 38 | 1564 | - | R | R | S | R | S | R | - | - | R | S | S | S | R | R | R | R | R | R | + |
|  | Bucharest | 39 | 395 | - | R | R | R | R | S | R | - | - | R | R | S | S | R | R | R | R | R | R | + |
|  | Bucharest | 43 | 101 | - | R | R | R | - | - | R | - | - | R | R | R | R | R | S | R | R | R | R | + |
|  | Galati | 60 | 17 | - | R | R | R | R | R | R | - | - | R | S | R | S | R | S | - | R | S | R | + |
|  | Bucharest | 86 | 101 | - | R | R | R | R | R | - | R | R | R | R | R | R | R | S | - | R | R | R | + |
|  | Bucharest | 87 | 258 | - | R | R | R | R | R | - | R | R | R | R | R | R | R | R | - | S | R | R | + |
|  | Bucharest | 89 | 101 | - | R | R | R | R | R | - | R | R | R | R | R | R | R | S | - | R | R | R | + |
|  | Targoviste | 120 | 101 | - | R | R | - | R | S | R | - | R | R | S | S | S | R | S | R | S | R | R | + |
|  | % of resistant strains/antibiotic | | | | 100 | 100 | 70 | 90 | 50 | 70 | 30 | 40 | 100 | 60 | 50 | 40 | 100 | 50 | 60 | 80 | 90 | 100 | 100 |

AMP = ampicillin, PRL = piperacillin, AMC = amoxicillin-clavulanic acid, CXM = cefuroxime, FOX = cefoxitin, CRO = ceftriaxone, CTX = cefotaxime, KZ = cefazolin, FEP = cefepime, ETP = ertapenem, IMP = imipenem, MEM = meropenem, ATM = aztreonam, GEN = gentamicin, AK = amikacin, CN = cefotetan, TET = tetracycline, CIP = ciprofloxacin, SXT = trimethoprim-sulfamethoxazole, MDR = multidrug resistant
