# Supplementary material for: Whole genome sequencing snapshot of multi-drug resistant Klebsiella pneumoniae strains from hospitals and receiving wastewater treatment plants in Southern Romania
Source: PLoS One. 2020 Jan 30;15(1):e0228079. doi: 10.1371/journal.pone.0228079 (PMC6992004; doi:10.1371/journal.pone.0228079)
Supplement: S2 Table — (DOCX) [file pone.0228079.s002.docx]

**Supplementary Table 2.** ARG frequencies in clinical and aquatic samples

| **Resistance genes** | **C (n=)** | **I (n=)** | **E (n=)** | **% C** | **% I** | **% E** |
| --- | --- | --- | --- | --- | --- | --- |
| **Antiseptics** | | | | | | |
| *oqxA* | 2 | 7 | 8 | **15.4** | **38.9** | **50.0** |
| *oqxA10* | 7 | 8 | 4 | **53.8** | **44.4** | **25.0** |
| *oqxA11* | 0 | 2 | 0 | **0.0** | **11.1** | **0.0** |
| *oqxA3* | 0 | 0 | 1 | **0.0** | **0.0** | **6.3** |
| *oqxA5* | 2 | 1 | 2 | **15.4** | **5.6** | **12.5** |
| *oqxA6* | 1 | 1 | 1 | **7.7** | **5.6** | **6.3** |
| *oqxB* | 2 | 7 | 8 | **15.4** | **38.9** | **50.0** |
| *oqxB11* | 0 | 1 | 1 | **0.0** | **5.6** | **6.3** |
| *oqxB12* | 0 | 1 | 1 | **0.0** | **5.6** | **6.3** |
| *oqxB13* | 0 | 5 | 1 | **0.0** | **27.8** | **6.3** |
| *oqxB14* | 0 | 1 | 0 | **0.0** | **5.6** | **0.0** |
| *oqxB17* | 7 | 4 | 3 | **53.8** | **22.2** | **18.8** |
| *oqxB19* | 3 | 1 | 1 | **23.1** | **5.6** | **6.3** |
| *oqxB20* | 0 | 3 | 1 | **0.0** | **16.7** | **6.3** |
| *qacE* | 1 | 0 | 0 | **7.7** | **0.0** | **0.0** |
| *qacEdelta1* | 4 | 12 | 12 | **30.8** | **66.7** | **75.0** |
| **Quinolones** | | | | | | |
| *qnrB1* | 3 | 0 | 1 | **23.1** | **0.0** | **6.3** |
| *qnrB10* | 0 | 0 | 1 | **0.0** | **0.0** | **6.3** |
| *qnrB19* | 0 | 0 | 1 | **0.0** | **0.0** | **6.3** |
| *qnrB36* | 0 | 0 | 1 | **0.0** | **0.0** | **6.3** |
| *qnrB4* | 0 | 0 | 1 | **0.0** | **0.0** | **6.3** |
| *qnrB67* | 0 | 0 | 1 | **0.0** | **0.0** | **6.3** |
| *qnrD1* | 0 | 1 | 0 | **0.0** | **5.6** | **0.0** |
| *qnrS1* | 1 | 5 | 5 | **7.7** | **27.8** | **31.3** |
| **ESBL & carbapenems** | | | | | | |
| *CMY4* | 0 | 5 | 1 | **0.0** | **27.8** | **6.3** |
| *CTXM15* | 10 | 11 | 11 | **76.9** | **61.1** | **68.8** |
| *DHA1* | 0 | 0 | 1 | **0.0** | **0.0** | **6.3** |
| *EC* | 0 | 1 | 0 | **0.0** | **5.6** | **0.0** |
| *EC15* | 0 | 1 | 0 | **0.0** | **5.6** | **0.0** |
| *KPC2* | 1 | 5 | 4 | **7.7** | **27.8** | **25.0** |
| *NDM1* | 1 | 5 | 2 | **7.7** | **27.8** | **12.5** |
| *OXA1* | 9 | 7 | 7 | **69.2** | **38.9** | **43.8** |
| *OXA10* | 0 | 5 | 1 | **0.0** | **27.8** | **6.3** |
| *OXA162* | 0 | 1 | 1 | **0.0** | **5.6** | **6.3** |
| *OXA48* | 6 | 5 | 7 | **46.2** | **27.8** | **43.8** |
| *OXA9* | 1 | 5 | 4 | **7.7** | **27.8** | **25.0** |
| *SHV1* | 1 | 0 | 1 | **7.7** | **0.0** | **6.3** |
| *SHV100* | 1 | 5 | 1 | **7.7** | **27.8** | **6.3** |
| *SHV101* | 1 | 3 | 2 | **7.7** | **16.7** | **12.5** |
| *SHV106* | 9 | 3 | 4 | **69.2** | **16.7** | **25.0** |
| *SHV107* | 0 | 1 | 0 | **0.0** | **5.6** | **0.0** |
| *SHV11* | 0 | 1 | 2 | **0.0** | **5.6** | **12.5** |
| *SHV12* | 2 | 5 | 3 | **15.4** | **27.8** | **18.8** |
| *SHV145* | 0 | 6 | 1 | **0.0** | **33.3** | **6.3** |
| *SHV158* | 2 | 6 | 7 | **15.4** | **33.3** | **43.8** |
| *SHV161* | 1 | 0 | 0 | **7.7** | **0.0** | **0.0** |
| *SHV187* | 4 | 8 | 5 | **30.8** | **44.4** | **31.3** |
| *SHV33* | 0 | 0 | 1 | **0.0** | **0.0** | **6.3** |
| *TEM1* | 7 | 9 | 10 | **53.8** | **50.0** | **62.5** |
| *TEM150* | 5 | 2 | 3 | **38.5** | **11.1** | **18.8** |
| **Aminoglycosides** | | | | | | |
| *aac(3)Iia* | 8 | 5 | 7 | **61.5** | **27.8** | **43.8** |
| *aac(3)IId* | 1 | 0 | 0 | **7.7** | **0.0** | **0.0** |
| *aac(6')Ib* | 0 | 0 | 1 | **0.0** | **0.0** | **6.3** |
| *aac(6')Ib'* | 2 | 3 | 4 | **15.4** | **16.7** | **25.0** |
| *aac(6')Ibcr* | 2 | 3 | 4 | **15.4** | **16.7** | **25.0** |
| *aac(6')Iid* | 10 | 12 | 11 | **76.9** | **66.7** | **68.8** |
| *aac(6')Il* | 0 | 5 | 1 | **0.0** | **27.8** | **6.3** |
| *aadA1* | 3 | 12 | 9 | **23.1** | **66.7** | **56.3** |
| *aadA2* | 1 | 6 | 7 | **7.7** | **33.3** | **43.8** |
| *aadA5* | 0 | 0 | 1 | **0.0** | **0.0** | **6.3** |
| *ant(2'')Ia* | 13 | 16 | 16 | **100.0** | **88.9** | **100.0** |
| *aph(3')Ia* | 2 | 0 | 3 | **15.4** | **0.0** | **18.8** |
| *aph(3'')Ib* | 6 | 9 | 6 | **46.2** | **50.0** | **37.5** |
| *aph(3')VI* | 0 | 5 | 1 | **0.0** | **27.8** | **6.3** |
| *aph(6)Id* | 6 | 9 | 6 | **46.2** | **50.0** | **37.5** |
| *rmtC* | 1 | 0 | 1 | **7.7** | **0.0** | **6.3** |
| **Others** | | | | | | |
| *tet(A)* | 2 | 1 | 6 | **15.4** | **5.6** | **37.5** |
| *tet(D)* | 6 | 4 | 2 | **46.2** | **22.2** | **12.5** |
| *catA1* | 2 | 6 | 7 | **15.4** | **33.3** | **43.8** |
| *catA2* | 0 | 5 | 2 | **0.0** | **27.8** | **12.5** |
| *catB3* | 9 | 7 | 7 | **69.2** | **38.9** | **43.8** |
| *cmlA5* | 0 | 5 | 1 | **0.0** | **27.8** | **6.3** |
| *fosA* | 9 | 12 | 8 | **69.2** | **66.7** | **50.0** |
| *fosA6* | 4 | 6 | 8 | **30.8** | **33.3** | **50.0** |
| *fosA7* | 1 | 0 | 0 | **7.7** | **0.0** | **0.0** |
| *mphA* | 2 | 6 | 8 | **15.4** | **33.3** | **50.0** |
| *mphE* | 0 | 5 | 1 | **0.0** | **27.8** | **6.3** |
| *msrE* | 0 | 5 | 1 | **0.0** | **27.8** | **6.3** |
| *arr2* | 0 | 5 | 1 | **0.0** | **27.8** | **6.3** |
| *arr3* | 0 | 0 | 1 | **0.0** | **0.0** | **6.3** |
| *dfrA1* | 1 | 1 | 3 | **7.7** | **5.6** | **18.8** |
| *dfrA12* | 2 | 6 | 7 | **15.4** | **33.3** | **43.8** |
| *dfrA14* | 10 | 9 | 7 | **76.9** | **50.0** | **43.8** |
| *dfrA17* | 0 | 0 | 1 | **0.0** | **0.0** | **6.3** |
| *dfrA7* | 1 | 0 | 0 | **7.7** | **0.0** | **0.0** |
| *sul1* | 4 | 12 | 12 | **30.8** | **66.7** | **75.0** |
| *sul2* | 6 | 9 | 6 | **46.2** | **50.0** | **37.5** |
| *armA* | 0 | 5 | 1 | **0.0** | **27.8** | **6.3** |
| *ble* | 1 | 5 | 2 | **7.7** | **27.8** | **12.5** |
