# Supplementary material for: Whole genome sequencing snapshot of multi-drug resistant Klebsiella pneumoniae strains from hospitals and receiving wastewater treatment plants in Southern Romania
Source: PLoS One. 2020 Jan 30;15(1):e0228079. doi: 10.1371/journal.pone.0228079 (PMC6992004; doi:10.1371/journal.pone.0228079)
Supplement: S3 Table — (DOCX) [file pone.0228079.s003.docx]

**Supplementary Table 3**. Virulence gene frequencies in clinical and aquatic samples

| **Virulence genes** | **C (n=)** | **I (n=)** | **E (n=)** | **% C** | **% I** | **% E** |
| --- | --- | --- | --- | --- | --- | --- |
| **Adherence** | | | | | | |
| *fimA* | 8 | 17 | 14 | **61.5** | **94.4** | **87.5** |
| *fimB* | 0 | 1 | 0 | **0.0** | **5.6** | **0.0** |
| *fimC* | 7 | 9 | 4 | **53.8** | **50.0** | **25.0** |
| *fimD* | 0 | 1 | 1 | **0.0** | **5.6** | **6.3** |
| *fimE* | 12 | 17 | 13 | **92.3** | **94.4** | **81.3** |
| *fimF* | 0 | 1 | 0 | **0.0** | **5.6** | **0.0** |
| *fimH* | 0 | 1 | 0 | **0.0** | **5.6** | **0.0** |
| *fimI* | 0 | 2 | 0 | **0.0** | **11.1** | **0.0** |
| **Iron acquisition** | | | | | | |
| *entA* | 13 | 18 | 16 | **100.0** | **100.0** | **100.0** |
| *entB* | 13 | 18 | 16 | **100.0** | **100.0** | **100.0** |
| *entC* | 0 | 1 | 1 | **0.0** | **5.6** | **6.3** |
| *entD* | 0 | 3 | 1 | **0.0** | **16.7** | **6.3** |
| *entE* | 13 | 18 | 16 | **100.0** | **100.0** | **100.0** |
| *entF* | 0 | 2 | 1 | **0.0** | **11.1** | **6.3** |
| *entS* | 13 | 18 | 16 | **100.0** | **100.0** | **100.0** |
| *fepA* | 13 | 18 | 16 | **100.0** | **100.0** | **100.0** |
| *fepB* | 13 | 18 | 16 | **100.0** | **100.0** | **100.0** |
| *fepC* | 13 | 18 | 16 | **100.0** | **100.0** | **100.0** |
| *fepD* | 13 | 18 | 16 | **100.0** | **100.0** | **100.0** |
| *fepG* | 13 | 18 | 16 | **100.0** | **100.0** | **100.0** |
| *fes* | 0 | 2 | 0 | **0.0** | **11.1** | **0.0** |
| *fyuA* | 8 | 16 | 14 | **61.5** | **88.9** | **87.5** |
| *iroN* | 0 | 1 | 0 | **0.0** | **5.6** | **0.0** |
| *irp1* | 8 | 16 | 14 | **61.5** | **88.9** | **87.5** |
| *irp2* | 8 | 16 | 14 | **61.5** | **88.9** | **87.5** |
| *iucA* | 0 | 1 | 0 | **0.0** | **5.6** | **0.0** |
| *iucB* | 0 | 1 | 0 | **0.0** | **5.6** | **0.0** |
| *iucC* | 0 | 1 | 0 | **0.0** | **5.6** | **0.0** |
| *iucD* | 0 | 1 | 0 | **0.0** | **5.6** | **0.0** |
| *iutA* | 0 | 2 | 0 | **0.0** | **11.1** | **0.0** |
| *ybtA* | 8 | 16 | 14 | **61.5** | **88.9** | **87.5** |
| *ybtE* | 8 | 16 | 14 | **61.5** | **88.9** | **87.5** |
| *ybtP* | 8 | 16 | 14 | **61.5** | **88.9** | **87.5** |
| *ybtQ* | 8 | 16 | 14 | **61.5** | **88.9** | **87.5** |
| *ybtS* | 8 | 16 | 14 | **61.5** | **88.9** | **87.5** |
| *ybtT* | 8 | 16 | 14 | **61.5** | **88.9** | **87.5** |
| *ybtU* | 8 | 16 | 14 | **61.5** | **88.9** | **87.5** |
| *ybtX* | 8 | 16 | 14 | **61.5** | **88.9** | **87.5** |
| **SECRETION SYSTEM - T6SS-III** | | | | | | |
| *ompA* | 13 | 18 | 16 | **100.0** | **100.0** | **100.0** |
| **Others** | | | | | | |
| *algW* | 0 | 2 | 0 | **0.0** | **11.1** | **0.0** |
| *aslA* | 0 | 1 | 0 | **0.0** | **5.6** | **0.0** |
| *astA* | 0 | 1 | 0 | **0.0** | **5.6** | **0.0** |
| *cheD* | 0 | 3 | 0 | **0.0** | **16.7** | **0.0** |
| *clfA* | 2 | 1 | 3 | **15.4** | **5.6** | **18.8** |
| *clfB* | 1 | 1 | 2 | **7.7** | **5.6** | **12.5** |
| *cseA* | 0 | 1 | 0 | **0.0** | **5.6** | **0.0** |
| *espL4* | 0 | 2 | 0 | **0.0** | **11.1** | **0.0** |
| *flgE* | 0 | 1 | 0 | **0.0** | **5.6** | **0.0** |
| *flgG* | 0 | 1 | 0 | **0.0** | **5.6** | **0.0** |
| *flgH* | 0 | 1 | 0 | **0.0** | **5.6** | **0.0** |
| *gmhA/lpcA* | 0 | 1 | 0 | **0.0** | **5.6** | **0.0** |
| *gspF* | 0 | 1 | 0 | **0.0** | **5.6** | **0.0** |
| *gspG* | 0 | 2 | 0 | **0.0** | **11.1** | **0.0** |
| *gspH* | 0 | 1 | 0 | **0.0** | **5.6** | **0.0** |
| *htpB* | 0 | 1 | 0 | **0.0** | **5.6** | **0.0** |
| *katB* | 0 | 1 | 0 | **0.0** | **5.6** | **0.0** |
| *lpxC* | 0 | 1 | 0 | **0.0** | **5.6** | **0.0** |
| *mgtB* | 13 | 18 | 16 | **100.0** | **100.0** | **100.0** |
| *mgtC* | 13 | 18 | 16 | **100.0** | **100.0** | **100.0** |
| *mtrD* | 0 | 1 | 0 | **0.0** | **5.6** | **0.0** |
| *rfaE* | 0 | 1 | 0 | **0.0** | **5.6** | **0.0** |
| *sdrC* | 1 | 1 | 2 | **7.7** | **5.6** | **12.5** |
| *sdrD* | 0 | 1 | 1 | **0.0** | **5.6** | **6.3** |
| *sdrE* | 1 | 1 | 3 | **7.7** | **5.6** | **18.8** |
| *tcpI* | 1 | 4 | 4 | **7.7** | **22.2** | **25.0** |
| *tssH5/clpV* | 1 | 1 | 1 | **7.7** | **5.6** | **6.3** |
| *waaA* | 0 | 1 | 1 | **0.0** | **5.6** | **6.3** |
| *xcpA/pilD* | 13 | 18 | 16 | **100.0** | **100.0** | **100.0** |
| *xcpR* | 13 | 18 | 16 | **100.0** | **100.0** | **100.0** |
| *yagV/ecpE* | 13 | 18 | 16 | **100.0** | **100.0** | **100.0** |
| *yagW/ecpD* | 13 | 18 | 16 | **100.0** | **100.0** | **100.0** |
| *yagX/ecpC* | 13 | 18 | 16 | **100.0** | **100.0** | **100.0** |
| *yagY/ecpB* | 13 | 18 | 16 | **100.0** | **100.0** | **100.0** |
| *yagZ/ecpA* | 13 | 18 | 16 | **100.0** | **100.0** | **100.0** |
| *ykgK/ecpR* | 13 | 18 | 16 | **100.0** | **100.0** | **100.0** |
