# Supplementary figures and images for: Whole genome sequencing snapshot of multi-drug resistant Klebsiella pneumoniae strains from hospitals and receiving wastewater treatment plants in Southern Romania
Source: PLoS One. 2020 Jan 30;15(1):e0228079. doi: 10.1371/journal.pone.0228079 (PMC6992004; doi:10.1371/journal.pone.0228079)

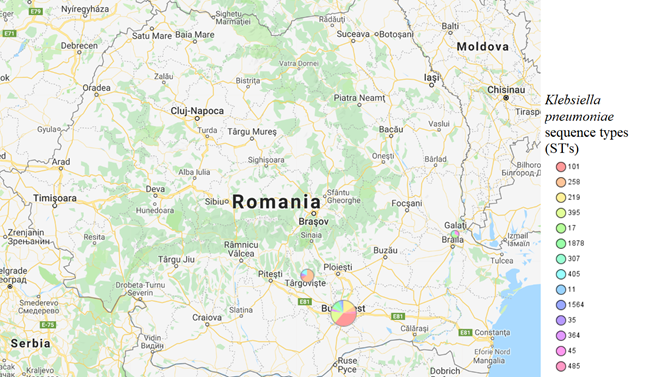

Supplement: S1 Fig — (TIF) [file pone.0228079.s004.tif]
